# LRRK2 Antisense Oligonucleotides Ameliorate α-Synuclein Inclusion Formation in a Parkinson’s Disease Mouse Model

**DOI:** 10.1016/j.omtn.2021.05.013

**Published:** 2021-05-29

**Authors:** Hien Tran Zhao, Neena John, Vedad Delic, Karli Ikeda-Lee, Aneeza Kim, Andreas Weihofen, Eric E. Swayze, Holly B. Kordasiewicz, Andrew B. West, Laura A. Volpicelli-Daley

(Mol Ther Nucleic Acids. *8*, 508–519; September 15, 2017)

In the originally published version of this article, in Figure 1B, the immunoblot of the LRRK2 protein for the ASO2 was inadvertently duplicated for ASO1. In Figure S2, the HSC70 loading control for the ASO1 was inadvertently duplicated for ASO2.

The figure panel images have been corrected to show the actual representative blots that were used for quantification. The authors regret this error.

**Figure 1:**
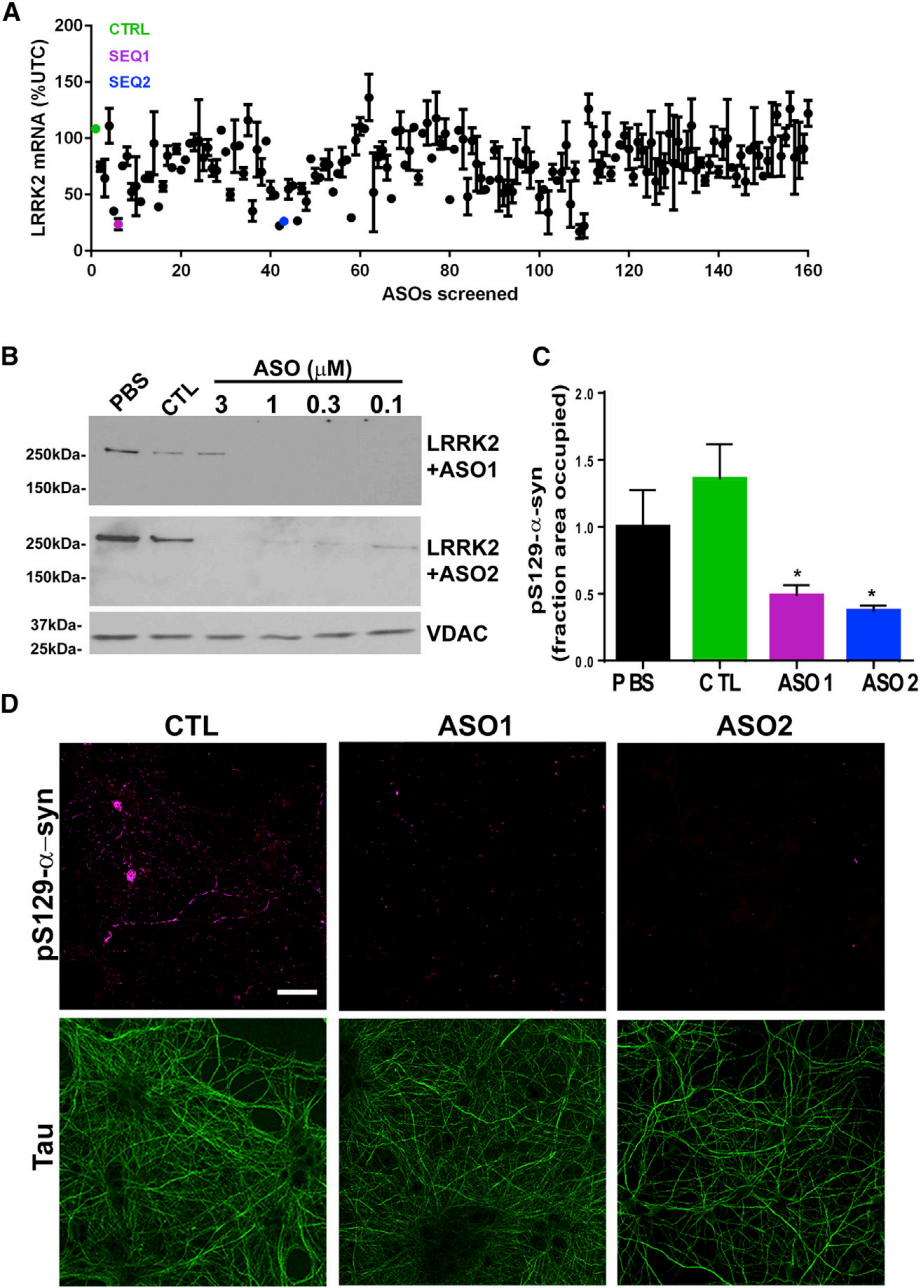
Identification of Efficacious LRRK2 ASOs that Reduce Formation of α-Syn Inclusions

**Figure S2:**
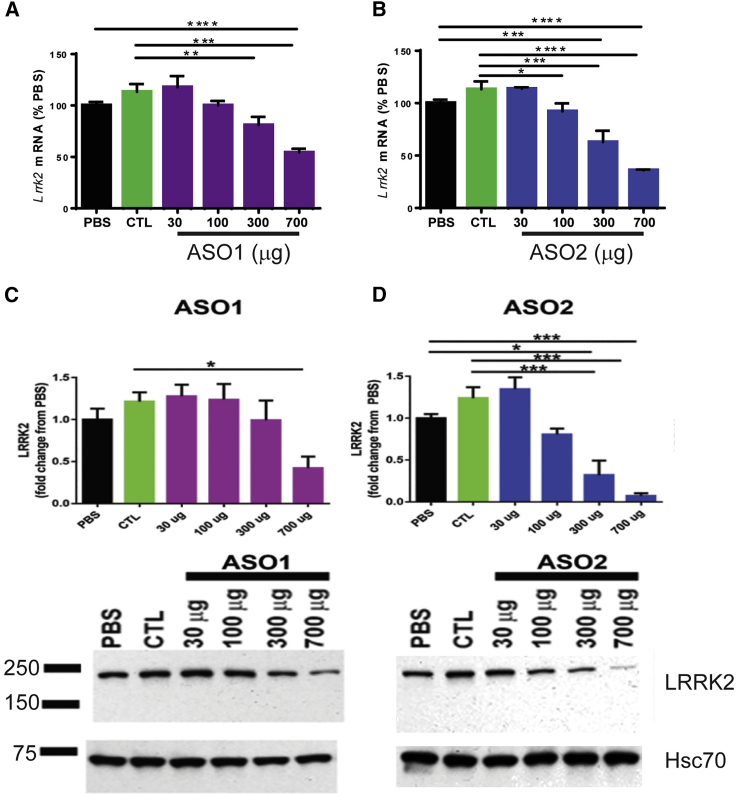
Dose-dependent reduction of Lrrk2 mRNA and LRRK2 protein in the cortex in LRRK2 ASO-treated mice

